# Applying the Principles of Bloom’s Taxonomy to Managing Tachyarrhythmia: Results of a Tachyarrhythmia Workshop

**DOI:** 10.7759/cureus.4037

**Published:** 2019-02-08

**Authors:** Ghulam Rehman Mohyuddin, Nicholas Isom, Laura Thomas

**Affiliations:** 1 Internal Medicine, University of Kansas Medical Center, Kansas City, USA

**Keywords:** medical education, tachycardia, simulation

## Abstract

Introduction

Resident physicians are routinely required to evaluate and manage patients with tachyarrhythmias. We developed a comprehensive workshop in an effort to improve residents’ competence and confidence at managing tachyarrhythmias.

Methods

A total of 55 residents attended the workshop and underwent pre- and post-testing to assess their competence of identifying and managing different arrhythmia. The participants were also asked to describe the comfort level managing these patients. After the pre-test, they participated in an interactive one hour session in which a cardiologist discussed common tachyarrhythmias and their management. For the next hour, the residents were then divided into groups of four to five. Using mannequins connected to heart monitors, the residents would be provided with a clinical vignette and asked to identify the heart rhythm and suggest management. A mock medication cart, and actual cardioverters-defibrillators were available. If the resident physician were to deliver cardioversion appropriately, the rhythm would change to sinus, and the patient’s hemodynamics would improve, thus providing live feedback for correct management.

Results

Amongst the 55 residents that participated in this study, the mean scores were 13.1 for pre-testing and 17.9 for post-testing, respectively (p = 0.0001). Residents’ mean comfort levels at managing tachyarrhythmias were 2.6 prior to testing and 3.6 post-testing (p = 0.0001).

Conclusion

We demonstrate that a two-hour focused tachyarrhythmia workshop significantly improved residents’ comfort level and competence in managing patients with tachyarrhythmia. By focusing on the higher levels of Bloom’s taxonomy such as analysis, synthesis and evaluation, we were able to improve the educational experience for our resident physicians.

## Introduction

Resident education is an area of continual investigation and experimentation, where innovative strategies are often being offered, however there is strong evidence in the literature to support case-based learning [1-3]. Case-based learning followed by a test to evaluate the learning in real time has been shown to increase resident engagement and involvement in the learning process [3]. Research in the education field has demonstrated a specific need for greater training in electrocardiography (EKG) interpretation for resident physicians [4, 5].

Case-based learning or problem-based learning has a growing amount of support for its efficacy in the literature since its development in 1965 [6]. It is identified not only as an effective tool for imparting new knowledge to the learner but also for monitoring learners over time and tracking their progress in self-directed learning [7]. With such an efficacious educational method, the goal is always utilizing it appropriately in order to maximize benefit.

Our curriculum was a focused trial aimed at improving resident competence at managing tachyarrhythmias. The need for such a curriculum was identified both by the program leadership and the residents themselves. Arrhythmias are a common encounter for a medicine resident and training in appropriate management is essential [4]. The goal was to develop a comprehensive simulation workshop centered on case-based learning and use of highly interactive simulated mannequins. The overall objective of the workshop was to enhance internal medicine resident’s ability to correctly identify and manage tachyarrhythmias, thus lending to increased confidence and independence in our training physicians.

There are a number of resources focusing on the education for a particular arrhythmia [8, 9]. However, our curriculum is unique in its focus on mastering the algorithmic processing of arrhythmias and using this to guide decision making.

## Materials and methods

Our curriculum participants were 55 internal medicine residents, whose training level spanned from post graduate year (PGY) 1 to PGY-3. The residents attended the workshop in eight separate groups.

At the start of the workshop all residents were pre-tested in order to assess their baseline comfort with the tachyarrhythmias. This pre-test consisted of five brief clinical vignettes with corresponding EKG strips for interpretation. The learners were then asked to identify the tachyarrhythmia and suggest management. All residents were also assessed on their comfort level at managing patients with arrhythmias using a questionnaire designed by the authors and validated by staff cardiologists.

After the pre-test, all residents then participated in an interactive one-hour educational session. In each group, a staff cardiologist discussed common tachyarrhythmias and their management with the residents. The discussion was accompanied with a set of slides both projected in the classroom and printed for note taking.

The residents were then divided into small groups of five learners. Each group went through a series of 10 clinical cases under the guidance of the attending. One staff physician was deemed appropriate to facilitate each smaller group of four to five residents. Using interactive mannequins (Gaumard HAL S3201 simulator) connected to heart monitors, the residents were provided with a clinical vignette and asked to identify the heart rhythm and proceed with their management. The mannequins used were able to produce various heart rhythms displayed on telemetry. Furthermore, they also responded to cardioversion, defibrillation and virtual medication. A medication cart and actual defibrillators/cardioverter (HeartMate) were available for the learners' use to fully simulate their management plan.

If the resident were to deliver the correct management, the rhythm on the telemetry would change to sinus rhythm and patient's hemodynamics would improve. In case of wrong management, there would be no improvement in the rhythm or vitals and the patient would likely decompensate. At this point, the staff physician would provide immediate feedback including the identification of the rhythm with the clinical scenario and the correct management. The resident would then have a second attempt managing the same rhythm to help reinforce the lesson learnt.

Our institution has a simulation center where defibrillators, mannequins, code carts, and simulated medications are available for educational purposes. Physicians volunteered their time to be instructors for the sessions. Simulation personnel to set up and program the equipment, as well as paper copies, were the only direct cost associated with this session. All other equipment is regularly used by our institution and available for these sessions.

As this was considered part of the standard curriculum, this study was considered exempt from institutional board review.

Following the lecture and exercise, residents completed an anonymous post-test similar to the pre-test. The tests were graded based on correct identification as well as management. A maximum of 20 points could be obtained on the test, 10 each for diagnosis and management of tachyarrhythmias respectively.

A statistical software, Statistical Package for the Social Science (SPSS, IBM, Armonk, NY) Version 24, was used to calculate statistics for this study. Paired sample t-tests were used to calculate p values.

## Results

The goal of the project was to assess whether or not this educational session improved the resident’s ability to identify and manage the tachyarrhythmias. The mean total scores were calculated for the pre-test and the post-test. These were scored out of the total 20 possible points for all 55 of the participating learners. The mean scores were found to be 13.1 with a standard deviation of 2.4 for pre-testing and 17.9 with a standard deviation of 4.0 for post-testing (p = 0.0001).

We further broke down the data to look specifically at the changes in diagnosis and management questions. The mean scores for the tachyarrhythmia management questions were 5.6 and 9.2 for pre-testing and post-testing, respectively (p = 0.0001). For questions relating solely to diagnosis, the mean scores were 7.5 and 9.0 for pre-testing and post-testing, respectively (p = 0.0001). This improvement in mean scores was statistically significant as well but represented a smaller improvement compared to that seen in the management category.

The scores were also broken down according to the PGY levels of all the participants. The participants were all internal medicine residents from PGY 1 to PGY 3. There were no statistically significant differences in test scores between the different PGY-levels.

The residents’ comfort level was also assessed in both the pre- and post-testing. The mean self-perceived comfort level at managing tachyarrhythmias was 2.6 prior to testing and 3.6 post-testing (p = 0.0001).

## Discussion

Our results help provide strong support for case-based learning and use of interactive mannequins that provide realistic live feedback for management approaches.

The development of this curriculum faced a number of obstacles to overcome; one of which was utilizing new simulation technology as effectively as possible. The Gaumard HAL S3201 simulator was the simulator used in our institution. These have a wide range of function and capability. The simulator is able to interact and respond to native equipment including, blood pressure cuffs, 12 lead EKG monitors, defibrillators and mechanical ventilators. Additionally the interactive software used to control the mannequin allows it to interact and respond to over 50 medications [10]. This feature allowed the learners to get real time feedback after selecting anti-arrhythmics to treat specific tachyarrhythmias.

The simulation was designed to be as realistic as possible by matching the mannequin’s reactions to the appropriate clinical condition. This provided the residents an opportunity to familiarize themselves not only with the standard of care management of arrhythmias, but also the HeartMate defibrillators used in practice at our institution. This in turn led to a greater sense of reality and gravity when making the decision to move forward with cardioversion in the simulation. This is supported by evidence in the literature that using realistic simulation versus traditional testing increases the participants’ physiologic stress response, with its resultant positive effects of cognition and learning [11].

In formulating this lesson plan, we hoped it would be a curriculum amenable to emulation and replication in the future for further successful education. During its creation Bloom’s taxonomy was kept in mind. Focus was placed on the upper tiers of this educational hierarchy, which includes those processes involved in critical thinking [12]. The curriculum engaged analysis, in that the residents had to identify the rhythm presented on the EKG by identifying various individual components of the rhythm and determine if those parts met the criteria for any specific diagnosis. Following the analysis, the learners then progressed to synthesis, the next level of the hierarchy. In the synthesis of the data provided, the learners had to incorporate the patient’s clinical condition in order to determine the best management of the patient. After moving forward with the plan, the learner had decided on, he or she then was able to receive live feedback regarding the outcome of the management selected. This is where the final stage of the critical thinking was engaged. At this point the learner was provided feedback as to how effective they perceived the session to be and the change in their comfort with the material. In this final stage, the educator then was able to use said feedback and reflect on the session to judge its value.

The study had a number of limitations. The first of which was the limited number of learners. While the number of residents involved within our program was maximized, the module was carried out in a single institution, thus the sample size was limited. Another limitation could be bias introduced by the repeat testing. A control group of residents taking a pre- and post-test without intermediary education may eliminate the possibility of some of this bias in future studies.

Future studies from this particular curriculum could be designed to compare this educational activity to a standard learning session. A more standard approach may be reading and testing thereafter. To ensure that our more costly approach is worthwhile, it would be important to know that it is not only effective, but also more effective than the traditional educational experience. Lastly, possible improvements to this particular module could include individualized simulation stations, or incorporate other heart rhythms such as bradyarrhythmias.

## Conclusions

Management of arrhythmias is an integral skill for internal medicine resident physicians to master. We demonstrate that a two-part focused tachyarrhythmia workshop significantly improved residents’ comfort level and competence in managing patients with tachyarrhythmia. By focusing on the higher levels of Bloom’s taxonomy such as analysis, synthesis and evaluation, we were able to improve the educational experience for our resident physicians, and we thus propose a model that can be replicated at other institutions.
